# The choice of alternatives to acute hospitalization: a descriptive study from Hallingdal, Norway

**DOI:** 10.1186/1471-2296-14-87

**Published:** 2013-06-22

**Authors:** Øystein Lappegard, Per Hjortdahl

**Affiliations:** 1 Department of Hallingdal Sjukestugu, Medical Clinic of Ringerike General Hospital, Vestre Viken Hospital Trust, Ål, Norway; 2 Department of General Practice, Institute of Health and Society, University of Oslo, Oslo, Norway

**Keywords:** General practice, Patient admissions, Referrals, Emergencies, Hospitalization, Community hospital, Intermediate care, Nursing homes

## Abstract

**Background:**

Hallingdal is a rural region in southern Norway. General practitioners (GPs) refer acutely somatically ill patients to any of three levels of care: municipal nursing homes, the regional community hospital or the local general hospital. The objective of this paper is to describe the patterns of referrals to the three different somatic emergency service levels in Hallingdal and to elucidate possible explanations for the differences in referrals.

**Methods:**

Quantitative methods were used to analyse local patient statistics and qualitative methods including focus group interviews were used to explore differences in referral rates between GPs. The acute somatic admissions from the six municipalities of Hallingdal were analysed for the two-year period 2010–11 (n = 1777). A focus group interview was held with the chief municipal medical officers of the six municipalities. The main outcome measure was the numbers of admissions to the three different levels of acute care in 2010–11. Reflections of the focus group members about the differences in admission patterns were also analysed.

**Results:**

Acute admissions at a level lower than the local general hospital ranged from 9% to 29% between the municipalities. Foremost among the local factors affecting the individual doctor’s admission practice were the geographical distance to the different places of care and the GP’s working experience in the local community.

**Conclusion:**

The experience from Hallingdal demonstrates that GPs use available alternatives to hospitalization but to varying degrees. This can be explained by socio-demographic factors and factors related to the medical reasons for admission. However, there are also important local factors related to the individual GP and the structural preparedness for alternatives in the community.

## Background

Traditionally, in Norway and most other Western countries, general practitioners (GPs) have two treatment options when dealing with acute health problems: to assume responsibility and liability for treating the patient at home or to refer the patient to hospital and thus transfer the treatment responsibility to hospital specialists. The number of acute admissions to hospitals is increasing in most Western countries. To curb expenses, health care providers in many countries are exploring viable alternatives to hospital admissions [1-3]. Some alternatives are based on enhanced care in the patient’s home given by the municipality’s home nursing service or with contributions from the specialist health care service, known as “hospital at home” [4]. Other alternatives include acute outpatient clinics or admissions to institutions at a lower level than the general hospital [5].

There are presently few nationally based alternatives to general hospital admissions in Norway. The National Coordination Reform, which is currently under implementation, challenges national health care providers to develop alternative medical services to reduce or replace general hospital admissions [6]. The present health care system in Norway is divided into two levels. The state has the responsibility for the specialist health services including the public hospitals and the outpatient services. The municipalities have the responsibility for the primary health services including emergency care, general practice, home-based care and nursing homes. GPs play a key role in the decision-making process regarding the level of acute hospitalizations. In Hallingdal, a rural region in southern Norway, a practice has developed over the past few years in which GPs can refer acutely ill patients to any of three different treatment levels: to a community-based nursing home, to a regionally based community hospital or to the nearest general hospital.

The objective of this paper is to describe the various patterns of admission to different somatic emergency service levels in Hallingdal and to discuss the reasons for the differences in utilization. An “acute patients’ steps” model has been developed to visualize the range of possible options available to Norwegian GPs treating acutely ill patients in the near future.

## Methods

Hallingdal comprises six municipalities (named A to F in the tables) with a total of 20,323 inhabitants (2011). Each of the six municipalities has a nursing home with its own administration and regulations regarding acute admissions and short-term beds. The medical supervision of the nursing home is performed by a local GP employed by the municipality as a nursing home doctor, devoting between 13 and 35 minutes per patient per week. In two of the municipalities (E and F), the nursing home doctor can be contacted by telephone out of hours, whereas the other four rely on voluntary medical services by other GPs in the municipality or on assistance from the GP on call in the region.

The nearest general hospital, Ringerike Sykehus (RS), is within 1–3 hours’ drive from the six municipalities. RS has a decentralized specialist health care service, Hallingdal Sjukestugu (HSS), located in the municipality of Ål, 170 km from the hospital. HSS can be described as a community hospital with a somatic inpatient unit with 14 beds, somatic and psychiatric outpatient clinics, a day treatment centre with dialysis and palliative care, a digital X-ray satellite to RS, and a base for helicopter and ground ambulances. The somatic inpatient unit at HSS fills a gap between the municipal health care services and the specialist health care at the hospital and can be categorized as an intermediate department. The use of HSS during the period 2009–10 has been described in a previous article [7]. The somatic inpatient unit at HSS had an average of 228 acute admissions a year with a mean length of stay of 3.8 days during this period.

Demographic data of the six municipalities of the Hallingdal region collected from official national statistics are listed in Table 1. The table includes data for the Health Care Needs Index (HCNI) [8]. This is a national governmental index weighting each municipality’s needs for specialist health care services based on age structure, indicators of morbidity, education level and other living conditions.

**Table 1:** Demographic and geographical data of the six municipalities in Hallingdal

**Municipality**	**Number of inhabitants**	**Inhabitants>80 years**	**Inhabitants>80 years per 100 inhabitants**	**Health care needs index**	**Distance in km to RS**	**Distance in km to HSS**
A	1000	83	8.3	1.18	85	77
B	3445	245	7.1	1.07	118	46
C	4572	264	5.7	1.04	137	25
D	2140	91	4.3	0.88	165	49
E	4713	313	6.6	1.05	162	0
F	4453	253	5.7	1.03	187	26
Sum	20,323	1249	6.1			

Total population and population >80 years of age in the six municipalities in Hallingdal. The Health Care Needs Index (HCNI) [8] values are reported relative to the national level, which is set at 1.0. Distances are from the municipality centre to Ringerike Sykehus (RS) and Hallingdal Sjukestugu (HSS).

This article is based on national hospital admission statistics for the years 2010–11 provided by the Department of Business data and finances, Vestre Viken HF. Further data were gathered from the six municipalities regarding doctor-initiated acute admissions to the local nursing homes in the period May 2010 to December 2011. Four of the municipalities already had such data, but two (B and C) did not have continuous statistics for the relevant period. The chief municipal medical officers of these two communities estimated the average number of patients who were referred acutely from the local doctors to the nursing home.

A focus group interview with the six chief municipal medical officers in Hallingdal was conducted to elucidate the practice of acute admissions to the different levels of the health care system [9]. During the past few years, these six officers, including the first author, have met once a month to discuss professional matters. One of these meetings was used to conduct the focus interview. The participants had been informed previously about the study, but this meeting was the first time they were presented with statistical findings from each municipality. The participation was voluntary and oral consent was obtained from all the participants in the group. The discussion was led by the first author in accordance with a predefined interview guide (Additional file 1), and the discussion was recorded digitally and transcribed and analysed later [10]. In this analysis key points were marked with codes and these codes were grouped into themes and main themes using Microsoft OneNote. A few quotes were chosen to illustrate some of the main themes. The chosen quotes can not be linked to any individual member of the group. The analytic approach was done by the main author and later discussed in detail among the two authors.

The study is part of a larger research project “Acute admissions to Hallingdal Sjukestugu” approved by Regional Committee for Medical and Health Research Ethics in Norway (ref. 2009/1300).

## Results

For each of the six municipalities, the annual average numbers of acute admissions to the RS, HSS and local nursing homes during the period 2010–11 are given in Table 2. The total number of admissions per 1000 inhabitants ranged from 61 to 111. When these results were weighted according to the Health Care Needs Index [8], the difference between the municipalities decreased to 69 to 104 admissions per 1000 inhabitants (Table 2).

**Table 2:** Acute admissions to the different levels of care

	**RS**		**HSS**		**Nursing homes**		**Sum**		
**Municipality**	**n (%)**	**Per 1000 inhabitants**	**n (%)**	**Per 1000 inhabitants**	**n (%)**	**Per 1000 inhabitants**	**n (%)**	**Per 1000 inhabitants**	**Corrected for HCNI**
A	80 (91)	80	3 (3)	3	5 (6)	5	88 (100)	88	75
B	336 (88)	98	23 (6)	7	24 (6)*	7	383 (100)	111	104
C	336 (87)	73	41 (11)	9	8 (2)*	2	385 (100)	84	81
D	115 (88)	54	5 (4)	2	11 (8)	5	131 (100)	61	69
E	292 (71)	62	90 (22)	19	28 (7)	6	410 (100)	87	83
F	284 (75)	64	44 (11)	10	52 (14)	12	380 (100)	85	83
Hallingdal	1443 (81)	71	206 (12)	10	128 (7)	6	1777 (100)	87	

Acute admissions of residents in each municipality in Hallingdal described as the annual average, percentage distribution and admissions per 1000 inhabitants to Ringerike Sykehus (RS), Hallingdal Sjukestugu (HSS) and local nursing homes for 2010–11. The total number of admissions per 1000 inhabitants was corrected for the Health Care Needs Index (HCNI) [8] (see Table 1). * Estimated figures.

For Hallingdal collectively, 1443 of the 1777 (81%) acute patients were admitted to the general hospital (RS), 206 (12%) to the intermediate department at HSS and 128 (7%) to the nursing homes (Table 2). The percentage of acute admissions at a level lower than the general hospital differed between the municipalities and had a range of 9% to 29%. Acute admissions to the local nursing homes ranged from 2% to 14%, and acute admissions to HSS ranged from 3% to 22%.

**Figure 1:**
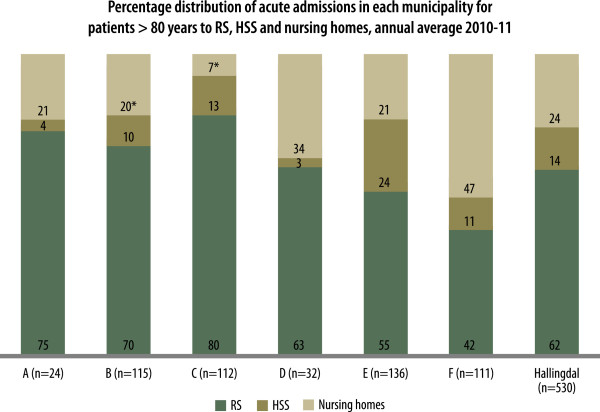
**Percentage distributions of acute admissions to the different levels.** Percentage distribution in each municipality of the number of acute admissions for patients >80 years to Ringerike Sykehus (RS), Hallingdal Sjukestugu (HSS) and local nursing homes, annual average for 2010–11. * Estimated figures.

Among the 1777 acute admissions, 530 were related to patients older than 80 years (30%). These ranged from 32 elderly of the 131 acute admissions in municipality D (24%) to 136 of 410 in municipality E (33%) (Table 2, Figure 1). Among these elderly patients, 62% were referred to RS, 14% to HSS and 24% to nursing homes (Figure 1). The percentage of acute admissions directly to the general hospital (RS) for this patient group differed between the municipalities, with a range of 42% to 80%. Similarly, the percentage of acute admissions to HSS ranged from 3% to 24% and to nursing homes from 7% to 47%.

In the qualitative part of the study, the members of the focus group reached a common understanding that the differences in the numbers of admissions between the municipalities depended largely upon the demographic and socio-economic variables of the inhabitants. The focus group members also noted that the patient’s clinical condition was a decisive factor in determining the level to which the patient was referred. Beyond these general reasons, the members identified a number of local contributing factors that influence the GP’s search for alternatives to hospitalization.

Regarding the variation in the use of HSS, the focus group emphasized the geographical distances from the municipality to the general hospital compared with that to the intermediate department. They noted that the distance was linked to the fact that the transport of patients from some of the municipalities to HSS is “up-stream” or counter-current to the direction of the hospital.

Public trust in the local health care system was another important contributor. The focus group participants perceived that the population of all six municipalities had great confidence in HSS. However, the public attitudes to acute admissions to local nursing homes were more variable. In municipalities where the inhabitants saw the nursing home mainly as a final refuge before death, it was more difficult for the population to accept acute referrals compared with those municipalities where the nursing home had developed, over time, a practice of short-term acute admissions. The focus group members also noted the patient’s trust in the individual GP as an important factor. Whereas experienced doctors often convey reassurance through admissions to local alternatives, similar solutions used by inexperienced or unfamiliar GPs often create insecurity among the patients and relatives.

The focus group members identified the referring GP’s years in practice and knowledge of the local health care services as key factors. This local knowledge covered a wide range, from organization and structure of the local health care facilities to detailed knowledge of an individual nurse’s capacity for handling acute admissions at the nursing home.

My impression is that younger doctors have a much greater tendency to refer patients to the hospital. They are not used to thinking like us, either they refer the patient to hospital or the patient is sent home.

The number of acute admissions to local nursing homes varied with the capacity and attitude of the staff. Nursing homes with available beds had more admissions than nursing homes where GPs constantly received responses indicating that all beds were occupied. They also remarked that the lack of available beds probably had more to do with the organization of the care services than with the capacity measured against the population.

The organization of the medical services at the nursing homes was a deciding factor. When the personnel at the nursing home were confident that a familiar doctor was available for phone consultations outside regular working hours, there was greater acceptance of admission of acute patients.

Formally, out-of-hour contacts are the responsibility of the doctor on call in the region, but it does not work like that in practice; we know the patients and we welcome these phone calls. A phone call can solve the problem within 30 seconds, and you know that if the nursing home calls the doctor on call, the patient will end up at the hospital. And the patient should never have been there.

Geographical proximity of the GP’s office to the nursing home was another key factor in the decision-making process.

If there are problems at the doctor’s office that are unresolved during the day, we refer to the nursing home. It is the same corridor; we roll the patient there in a wheelchair — very, very convenient.

## Discussion

The number of acute admissions varies over time. This study used data for small municipalities over a relatively short period of two years. The results were compared with official statistics on admissions to RS and HSS during 2004–11; no significant variations were found in the admissions pattern over these years. Data collected on acute admissions to the municipal nursing homes are not part of national statistics and are more uncertain. They should thus be taken more as an expression of tendencies than facts. Today, there are no other sources for these figures in Norway.

A focus group interview with the six chief municipal medical officers was chosen as the preferred qualitative method because its members are experienced GPs in clinical practice, including on-call duties, and thus possess first-hand knowledge of the use of the health care system in this region. In addition, they are, as chief municipal medical officers, expected to have an updated overview of the health services available in their communities. Because the first author is a regular member of the group, he may have influenced the group’s discussions. However, the members of the group have met for several years, are well acquainted and discuss these topics freely, and any influence of the first author is expected to be of lesser importance.

The literature shows that the frequency of acute admissions to hospitals differs between GPs and between municipalities [11,12]. However, we have found no reports that compared the rates of acute referrals to different *levels* of health care. Our study shows that alternatives to hospitalization are especially relevant for elderly people. In one municipality (F), 58% of the acute admissions of patients older than 80 years were to local alternatives. Some have warned against admissions to a lower level than a hospital for elderly patients with acute and severe loss of functions because of the fear of inadequate diagnosis or treatment [13]. Older people often value proximity to family and treatment in their own local surroundings compared with that provided by a hospital [7].

Referral rates do not necessarily indicate the quality of medical practice. In a literature review, O’Donnell summarized the causes of variation in referral rates into four major groups: a) patient-related factors, b) disease-related factors, c) GP-related factors, and d) structural factors [14].

Most of the variations linked to patient-related factors are socio-demographic characteristics such as age distribution, educational level, rates of chronic illness and rates of single-occupant households [12]. In our study, adjusting for the Health Care Needs Index also indicated the relevance of socio-demographic factors (Table 2).

The focus group members emphasized the disease-related factors and the patient’s medical condition as primary determinants when deciding where to refer patients. They also noted the importance of the existence of alternatives to hospitalization, when appropriate.

GP-related factors, such as expertise and interests, tolerance for uncertainty and an ability to deal with conflicting opinions from patients, relatives and other health professionals, also influence referral rates [15]. In the literature, job experience is not a factor linked to a doctor’s hospitalization pattern. However, the focus group members identified local work experience as an important factor in the doctor’s ability to use the three different levels. Hallingdal has an emergency health care system handled almost exclusively by local regular GPs with a few locum doctors. This is somewhat unusual compared with the rest of Norway. Locums will often tend to “play it safe” and refer acute patients to the highest level of care. It is a paradox that the present trend in Norway towards combining smaller on-call districts into larger inter-municipal emergency care units, where each doctor has less local working experience, may thus have resulted in a greater tendency towards hospital admissions, in contrast to the health authority’s recommendations [6].

Structural factors are important in relation to the differences in admission patterns. The focus group members noted that the location of the nursing home in relation to the GP’s office was important. In the municipality where the nursing home was used most frequently (F), the doctor’s office and the nursing home were located next to each other. This made co-operation and follow-up of patients easier. This closeness and collaboration can also partly explain why this municipality had more acute admissions of elderly patients to the local nursing home than acute admissions to HSS, which was 26 km away. Nevertheless, the combined number of acute admissions from this municipality was similar to the average number for Hallingdal.

It is difficult to make general recommendations about when and to which level acute patients should be referred [7]. Diseases develop gradually, and clinical diagnoses often overlap in symptoms and severity. Thus, the doctor must continuously combine disease progression and risk factors with knowledge of the individual patient when deciding when and where to refer the patient. With this degree of variability and uncertainty, one can expect a certain number of inappropriate admissions. International studies indicate that about 20% of admissions to a hospital could have been treated at a lower level [5,16,17]. Discussions about inappropriate admissions quickly target the patients who should have been referred to a higher level. Equally important may be those cases where patients are inappropriately admitted to a too-high level of care, creating unnecessary burdens on patients, hospitals and society.

**Figure 2:**
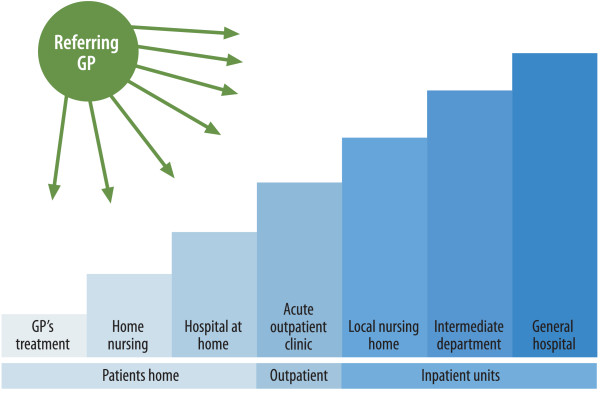
**The acute patients’ steps.** Referring GP’s options in the treatment of acute patients.

Various strategies to influence GPs’ admission profiles have been assessed [11,12,14]. The development of professional guidelines appears to have had little effect on admission practice, whereas measures to stimulate dialogue between GPs and hospital specialists have yielded encouraging results and are sought by GPs [18,19]. In an acute situation, the GP must quickly decide whether the patient should be hospitalized or given alternative services. Identification and clarification of options can make the GP better equipped to make the decision. In Figure 2, we introduce the “Acute patients’ steps” as an illustration of the different options the GPs of Hallingdal may have available in the near future. The different steps of the stairway illustrate the distinction between the services given at the patient’s home and the various types of health care services or admissions at the GP’s disposal. The GP must assess the appropriate level for an acutely ill patient at a given time and understand the patient’s medical needs, social situation and the different services available.

## Conclusion

Experiences from Hallingdal, Norway, demonstrate that there are wide variations in GPs’ use of alternatives to acute general hospitalization. Geography, characteristics of doctors and structural conditions are contributing local factors explaining the differences in utilization of these services. With the national efforts introducing the Coordination Reform, this experience may be a model for other Norwegian regions to explore and may have relevance internationally. The Hallingdal model has been developed locally. Further research and experience are needed to demonstrate its generalizability, quality, efficacy and sustainability.

## Abbreviations

GP: General practitioner; HCNI: Health care needs index, an official Norwegian national governmental index weighting each municipality’s needs for specialist health care services; HF: Helseforetak, hospital trust. Vestre Viken HF is one of ten hospital trusts owned by the South-Eastern Norway Regional Health Authority; HSS: Hallingdal Sjukestugu, a community hospital in Ål, Hallingdal; RS: Ringerike Sykehus, a general hospital in Hønefoss, 170 km. from Ål.

## Competing interests

The authors declare that they have no competing interests.

## Authors’ contributions

The authors alone are responsible for the content and writing of the paper. ØL is the main author. The article is part of a PhD study at the University of Oslo. PH has as a supervisor contributed with analysis, drafting and revising of the manuscript and has given the final approval. Both authors read and approved the final manuscript.

## Authors’ information

ØL is the chief municipal medical officer in the municipality of Ål, Hallingdal and also a researcher employed by RS. PH is a professor at the Department of General Practice, Institute of Health and Society, Medical Faculty, University of Oslo.

## Pre-publication history

The pre-publication history for this paper can be accessed here:

http://www.biomedcentral.com/1471-2296/14/87/prepub

## Supplementary Material

Additional file 1 **Focus group.** The chief municipal medical officers of the six municipalities in Hallingdal. Click here for file
