# Supplementary material for: The choice of alternatives to acute hospitalization: a descriptive study from Hallingdal, Norway
Source: BMC Fam Pract. 2013 Jun 22;14:87. doi: 10.1186/1471-2296-14-87 (PMC3698089; doi:10.1186/1471-2296-14-87)
Supplement: Additional file 1 — Focus group. The chief municipal medical officers of the six municipalities in Hallingdal. [file 1471-2296-14-87-S1.docx]

# INTERVIEW GUIDE

## Focus group: The chief municipal medical officers of the six municipalities in Hallingdal

### Informal introduction

- The moderator informs about the research project.

### Formal introduction

- 1. The moderator presents the focus group method.
- The participation is voluntary and it is possible to withdraw at any moment.
- A predefined interview guide is used as a basis for the discussion.
- The interview will be taped. Quotes from the interview can be used in an article, but quotes cannot be linked to individuals.
- The timeframe for interview is one hour.
- Consent is obtained from all the participants.
  1. The moderator presents the background and the objectives of the interview
- The moderator distributes statistics for acute admissions to RS, HSS and the local nursing homes for 2010-11.
- The moderator presents briefly the statistics and the differences that emerge between the municipalities.
- The aim of the focus group is to discuss factors that have an influence on the numbers of admissions to the three different levels of acute care.

### The tape recorder is put on

### The focus group discussion

Open conversation in the group. The moderator ensures that the following predefined main themes are discussed if they have not already been mentioned by the group:

- Demographics
- Medical diagnosis
- Geography and distance
- Location of the physicians’ offices in relation to the nursing homes
- Nursing home capacity and organization
- Organization of the work of the nursing home doctors
- Expertise on all levels
- Physicians' experience and local knowledge
- Different treatment cultures at various medical offices
- Reputation in the population according to the different inpatient locations
- Economic impact for the municipalities in various inpatient locations

### Summary

- The moderator summarizes and the focus group has the possibility to comment on this.
- Does anyone in the group feel that they possess opinions, perceptions or nuances that have not been elucidated?

### Finishing

- The moderator informs about further work in the research project and the expected use of the material.

### Short evaluation
